# HIV risk and influence factors among MSM who had sought sexual partners in core venues: a continuous sentinel surveillance in 2010–2022

**DOI:** 10.3389/fpubh.2024.1476642

**Published:** 2024-12-16

**Authors:** Zijie Yang, Lan Wei, Zhongliang Xu, Simei Li, Yiwen Xing, Yan Zhang, Yuan Yuan, Shaochu Liu, Wei Xie, Wei Tan, Wei Ye, Jingguang Tan, Xiangdong Shi, Xiangyu Yan, Tiejian Feng, Zhongwei Jia, Jin Zhao

**Affiliations:** ^1^School of Public Health, Peking University, Beijing, China; ^2^Shenzhen Center for Disease Control and Prevention, Shenzhen, China; ^3^Nanshan Center for Disease Control and Prevention, Shenzhen, China; ^4^School of Public Health, Sun Yat-sen University, Guangzhou, China; ^5^School of Public Health, Shanxi Medical University, Taiyuan, China; ^6^School of Disaster and Emergency Medicine, Tianjin University, Tianjin, China; ^7^Center for Intelligent Public Health, Institute for Artificial Intelligence, Peking University, Beijing, China; ^8^Center for Drug Abuse Control and Prevention, National Institute of Health Data Science, Peking University, Beijing, China; ^9^Peking University Clinical Research Institute, Beijing, China

**Keywords:** HIV/AIDS, men who have sex with men, venues network, sexual contact networks, HIV risk

## Abstract

**Background:**

Seeking sexual partners in men who have sex with men (MSM) venues has been regarded as a high-risk behavior for HIV among MSM. Nevertheless, with the implementation of venue-based interventions and the change in the way MSM seek sexual partners, the continued status of MSM venues as the HIV risk factor remains inconclusive. This study endeavors to delve into this ambiguity by examining the MSM sexual contact network (SCN) as a foundation.

**Methods:**

A series of cross-sectional surveys were conducted in Shenzhen in the period 2010–2022. MSM sexual contact network and venue network were acquired, and network metrics were employed to identify core MSM and core venues. We compared the risk of HIV and risk behaviors between MSM who sought sexual partners in core venues and those who did not, with subgroup analyses based on different time periods.

**Results:**

The overall HIV prevalence among the 4,408 MSM surveyed in this study was 14.6%. Notably, 17 core venues were identified out of the 68 reported MSM venues, with 1,486 MSM who had sought sexual partners in core venues. These MSM had significantly higher risk of HIV and were more likely to take HIV testing and receive intervention services. Subgroup analyses showed that the heightened HIV risk associated with seeking partners in core venues was specific to the period 2010–2014, while HIV testing and service access remained consistently higher across all-period subgroups. Multiple sexual partners, seeking partners in core venues, receptive or both sexual roles, drug abuse, absence of HIV test, unprotected anal intercourse (UAI), and lower education levels were associated with elevated HIV risk among MSM.

**Conclusion:**

Following the implementation of differentiated venue-based interventions, the risk of HIV among MSM who had sought sexual partners in core venues decreased to a level comparable to that of MSM who had not. The accessibility of HIV testing and intervention services remains uneven between MSM who had sought sexual partners in core venues and those who had not. As the Internet sex-seeking behavior gains prevalence among MSM, strategic adjustments of public health resource allocation may be necessary to address this imbalance.

## Introduction

Men who have sex with men (MSM) represent a demographic group at particularly high risk of contracting HIV. A previous study has been indicative that there is an estimated 8–12 million MSM in China (1). The proportion of newly diagnosed HIV infected attributed to male-to-male sexual transmission in China has significantly increased in the previous decade—from 12.6% in 2011 to 25.6% in 2022 (2). Notably, in 2022, over 60% of the newly diagnosed HIV infected from the developed cities in China were due to male-to-male transmission (3). Furthermore, the HIV prevalence among MSM in China increased from 4.9% in 2008 to 7.9% in 2020 (4). However, only approximately 68.9% of people living with HIV (PLWHA) in China are currently aware of their infection status (5). Therefore, it is essential to explore the factors driving HIV transmission within the MSM community, which could be helpful in identifying more undiagnosed cases of HIV and effectively mitigate the spread of the virus within MSM.

Currently, for controlling the HIV epidemic among MSM in China, MSM venue-based interventions are one of the primary methods. Before the increase in the popularity of MSM social applications (APPs), MSM social venues were the primary place where MSM sought sexual partners. Previous studies indicated that MSM who frequented venue-based socializing locations were among the subgroups that had the highest risk of contracting HIV (6, 7). Thus, these MSM venues might have played a major role in driving HIV transmission among MSM before the emergence of MSM APPs. Nevertheless, few studies in China have examined the impact of MSM venues on HIV transmission among MSM within sexual contact networks (SCN) among MSM. Variations in the likelihood of contracting HIV among individuals, who are vulnerable to HIV infection, are associated with the network structure (8). The composition of these networks significantly influences MSM’s HIV-related risk behaviors—it catalyzes HIV transmission and other sexually transmitted infections (STIs) (9). In addition, a previous study suggested that the social network-derived HIV transmission risk assessments exhibited strong correlations with HIV risk behaviors and outcomes in MSM, playing a crucial role in pinpointing high-risk individuals (10). This study aimed to evaluate the risk of HIV among MSM who had sought sexual partners in core MSM venues within MSM’s SCN and to explore the influence factors of HIV infection based on MSM SCN.

## Methods

### Data collection

Based on a series of cross-sectional surveys conducted in Shenzhen during the period 2010–2022, this study used the respondent-driven sampling (RDS) survey method to collect samples, with methodology details having been previously outlined (11). The study used the same sample selection criteria and the same questionnaire content for each year between 2010 and 2022. Data collection was conducted using electronic questionnaires on tablets, and unqualified questionnaires—including the questionnaires that did not meet the sample selection criteria, the questionnaires with low credibility, and the questionnaires that were incomplete, were automatically excluded by the predesigned system.

Relying on HIV voluntary counseling and testing (VCT) centers owned sites within MSM by non-governmental organizations, we recruited nine MSM of different ages, educational backgrounds, occupations, and monthly incomes who were willing to cooperate, had strong communication skills, and were supportive of the survey as initial participants (i.e., “seeds”). By providing specific economic incentives to participants, the “seeds” distributed RDS recruitment cards with unique serial numbers (RDS code) to other MSM, inviting their friends to come to a designated location to complete a questionnaire and get HIV testing. The MSM who accepted the survey were then asked to distribute the recruitment cards to other MSM they knew, and this referral chain continued until no new participants could be recruited. All potentially eligible individuals were invited to participate in our survey, which entailed the completion of a self-administered questionnaire and HIV testing. The questionnaire included inquiries about sociodemographic and HIV-related risk behaviors, as well as the provision of an RDS code and the type of relationship with the recommender (regular sex partners, casual sex partners, casual friends, Internet friends, and strangers), except for the nine seeds recruited by us annually. Each participant was required to provide a blood sample with a unique finger identity (ID) for HIV testing. Informed consent was obtained from all individuals prior to participation, ensuring the protection of their privacy. Participants were also asked to mention the venues that they usually visited for seeking sexual partners. To protect the interests of businesses, all MSM venues were analyzed using a venue code instead of a venue name, and the venues were divided into four main categories: bars, saunas, parks, and others.

The inclusion criteria for study participation were as follows: (1) Biologically male (2) aged 16 years or older (3) had engaged in sexual activity with men within the past 6 months, and (4) had no serious psychological disorders and possessed the ability to complete the survey independently.

### Data management

Shenzhen has started to implement venue-based differentiated behavioral interventions, which were offering venue-specific lubricant and condom services and design interventions tailored to the demographic characteristics and behaviors of clients in different venues, in all MSM venues from the end of 2014, which could influence the role of MSM venues in facilitating HIV transmission among MSM. The outbreak of the coronavirus disease 2019 (COVID-19) pandemic in 2020 led to the temporary closure of the majority of the MSM venues as part of epidemic prevention and control measures, further impacting their role in HIV transmission. To better identify the factors influencing HIV transmission in the sexual contact networks of MSM at different stages of the disease, the data were divided into three subsets for subgroup analyses by the time of implementation of the intervention and the time of the COVID-19 outbreak as the time cut-off points: 2010–2014, 2015–2019, and 2020–2022.

In a series of cross-sectional surveys, the same individual may participate in multiple surveys in different years. Therefore, after the segregation of the data, we used the respondent’s fingerprint information to identify various questionnaires and the HIV test results for the same MSM individual in a year group who participated in the survey in different years. For the analysis, we chose the questionnaire and HIV test results from the latest time in the survey for this year’s group. For the analysis of the period 2010–2022, in addition to conducting subgroup analyses, we also created an overall SCN; during these 13 years of investigation, we used the latest questionnaire data and HIV test results from these individuals.

### Definition and measure

#### HIV services

Participants received free HIV-related testing counseling, knowledge education, condom distribution, and other interventions from the Chinese Center for Disease Control and Prevention (CDC) or non-governmental organizations (NGOs) in the past 12 months.

#### Unprotected anal intercourse (UAI)

UAI was defined as respondents who had never or sometimes used condoms in anal intercourse with males in the past 6 months.

#### Drug abuse

The questionnaire also assessed the respondents’ past drug abuse behavior, which involved nine types of drugs (heroin, marijuana, cocaine, methamphetamine, ketamine, lysergic acid diethylamide, 3,4-methylenedioxymethamphetamine [MDMA], flunitrazepam, and cough drops [abuse]).

#### STIs

In a list of eight sexually transmitted diseases (syphilis, condyloma acuminatum, genital herpes, gonorrhea, genital *chlamydia trachomatis*, urethritis, pubic lice, and hepatitis), for each item, the participants were asked if they had ever been diagnosed for in the past 12 months. A dichotomous variable was calculated to represent infection of at least one STI in the past 12 months (“yes” or “no”).

#### HIV testing

Participants were asked whether they had ever been tested for HIV; those tested for HIV were then asked if they had been tested in the past 12 months.

#### Men who have sex with men and women (MSMW)

Respondents were asked if they have had sex with a woman in the last 6 months, and those who answered “Yes” were considered MSMW.

#### Money boy (MB)

Respondents were asked if they had had sex with other men in the past year to get money, and if they answered “Yes,” they were defined as MB.

#### HIV nearby

After observing whether MSM’s neighboring nodes in SCN are PLWHA or not; if one or more neighbors are PLWHA, then it is defined as HIV nearby.

#### HIV test nearby

After observing whether the neighboring nodes of the MSM in the SCN are those that have had an HIV test in the last 12 months; if one or more neighbors have not been tested, it is defined as HIV test nearby.

#### MB nearby

After observing e whether MSM’s neighboring nodes in SCN are MB or not; if one or more neighbors are turned to be PLWHA, then it is defined as MB nearby.

#### MSMW nearby

After observing whether MSM’s neighboring nodes in SCN are MSMW or not, if one or more neighbors turned to be MSMW, then it is defined as HIV nearby.

#### UAI nearby

After observing whether MSM’s neighboring nodes in SCN are UAI or not; if one or more neighbors are turned to be affected by UAI, then it is defined as UAI nearby.

#### Drug abuse nearby

After observing whether MSM’s neighboring nodes in SCN are drug abusers or not; if one or more neighbors are turned to be affected by drug abuse, then it is defined as drug abuse nearby.

#### Core MSM (venues)

In this study, the Technique for Order Preference by Similarity to an Ideal Solution (TOPSIS) was used to calculate the importance score of nodes in the network based on their degree centrality, betweenness centrality, closeness centrality, and eigenvector centrality in the network. The nodes with the top 25% of the score were defined as core nodes (12). In the MSM sexual contact network, a core node signifies an MSM individual who was sexually active. In the venue network, a core venue represents the establishment with the highest MSM population turnover and was predominantly frequented by MSM venues. Due to the constraints imposed by the RDS survey methodology, MSM participants were unable to nominate all their sexual partners for participation in the study. Consequently, we introduced dummy nodes for each MSM participant when assessing the degree centrality, betweenness centrality, closeness centrality, and eigenvector centrality. This adjustment was based on the number of sexual partners reported by the MSM in the last 6 months, to reflect their significance within the network accurately.

#### MSM who had sought sexual partners in core venues

The list of MSM venues where MSM reported in the questionnaire that they had sought sexual partners included core venues.

#### Assortative

To assess patterns of mixing by PLWHA and another demographic and behavioral characteristic of MSM in the primitive sexual contact network with no additional virtual nodes, we compared the proportion of contacts in the network for MSM. We also quantified assortative mixing with the assortative coefficient (*r*). Assortative coefficients range from −1 to 1. An assortative value of 1 signifies that nodes within the network exclusively connect with nodes sharing similar characteristics. A value of −1 indicates that nodes only link with nodes of differing factors. A value of 0 suggests that nodes connect randomly with a mix of similar or differing characteristics (13, 14).

### Sexual contact networks (SCN)

In the process of capturing MSM’s sexual contact networks, social association data were extracted to capture MSM’s sexual contact networks, regardless of how many surveys they participated in during the survey period.

#### Edge acquisition

In our survey, all respondents except the annual seed must fill in the questionnaire with the RDS code for them. We used the RDS codes to find the upstream respondents who had recommended their participation in our survey and to access the initial social networks of the MSM who participated.

#### Edge inclusion and exclusion

As mentioned earlier, all respondents were asked about their relationship with upstream respondents. If respondents answered that they were in a regular or occasional sexual partner relationship with the upstream respondent, the edge between them was included. If they answered that their relationship with the upstream respondent was a non-sexual contact relationship, the edge between them was excluded.

#### Node inclusion and exclusion

After obtaining the initial social network of the MSM, the degree of the nodes in the network is calculated; nodes with degree ≥ 1 were included, and nodes with degree = 0 were excluded.

### Network of MSM venues

We queried respondents on MSM venues visited for sexual partnering in the past year. If multiple venues were reported, we interconnected these venues in our network. For instance, if an MSM individual stated visiting A or B venues, we linked A–B in the venue network. Here, edges signify MSM mobility across venues. To capture the frequency of such movements, based on aggregated MSM venue mobility data, we calculated weighted edges as follows: $\mathrm{weight}=\frac{e_{ij}}{\sum e}$ , where *e_ij_* denotes the count of MSMs moving between the venues *i* and *j*.

### Statistical analysis

To analyze sociodemographic and behavioral data for all participants; descriptive statistics and *χ*^2^ tests were used. The variables with *p* < 0.1 in the univariate analysis were included in the multivariate logistic regression analysis. The multilevel logistic regressions were then used to evaluate the risk factors for HIV among MSM. The best model was identified by adopting forward selection variables. The statistical analysis used Python (version 3.11, Python Software Foundation) and Statistical Product Service Solutions (SPSS) (version 20.0, International Business Machines Corporation). Gephi (version 0.10.1, Gephi Consortium) was used to map the SCN; we mapped a three-layer network with three layers including venues, MSM who had sought sexual partners in core venues, and MSM who had not been seeking sexual partners in core venues. The data are presented as numbers (percentages), means ± standard deviations, adjusted odds ratios (aORs), and 95% confidence intervals (95% CIs). The significance level was considered to be 0.05.

## Results

### Overall sexual contact network in 2010–2022

A total of 4,408 MSM were enrolled in the SCN, 14.6% (643/4408) of whom were PLWHA, and 68 MSM venues were enrolled in the MSM venues network (Appendix Figure 1). 17 MSM venues were defined as core venues (Appendix Table 1), and 1,486 (33.71%, 1486/4408) MSM were reported ever seeking sexual partners at these venues. The demographic and behavioral characteristics comparative analysis among PLWHA and non-PLWHA is shown in Table 1. The comparative analysis of demographic and behavioral characteristics of MSM who had sought sexual partners in core venues and MSM who had not sought sexual partners in core venues is demonstrated in Table 2.

**Table 1:** A comparative analysis of the demographic and behavioral characteristics of PLWHA and non-PLWHA.

	Overall (2010–2022)			2010–2014			2015–2019			2020–2022		
	non-PLWHA (*n* = 3,765)	PLWHA (*n* = 643)	*χ*^2^/*F*	non-PLWHA (*n* = 1794)	PLWHA (*n* = 382)	*χ*^2^/*F*	non-PLWHA (*n* = 1,659)	PLWHA (*n* = 230)	*χ*^2^/*F*	non-PLWHA (*n* = 547)	PLWHA (*n* = 72)	*χ*^2^/*F*
Educational levels			56.14^§^			19.4^§^			37.33^§^			1.31
Junior high school and below	433 (77.3)	127 (22.7)		246 (75.5)	80 (24.5)		172 (80.4)	42 (19.6)		40 (83.3)	8 (16.7)	
High middle school	1,014 (82.8)	211 (17.2)		631 (81.0)	148 (19.0)		345 (84.4)	64 (15.6)		106 (88.3)	14 (11.7)	
College and above	2,305 (88.5)	300 (11.5)		915 (85.6)	154 (14.4)		1,148 (92.1)	99 (7.9)		401 (88.9)	50 (11.1)	
Drug abuse			11.6^§^			2.76*			6.68^§^			10.08^§^
Yes	473 (80.9)	112 (19.1)		93 (76.9)	28 (23.1)		355 (85.5)	60 (14.5)		102 (80.3)	25 (19.7)	
No	3,281 (86.2)	526 (13.8)		1701 (82.8)	354 (17.2)		1,310 (90.0)	145 (10.0)		445 (90.4)	47 (9.6)	
Number of sexual partners			31.04^§^			17.2^§^			5.58*			7.21^§^
1	1,652 (88.3)	219 (11.7)		770 (86.2)	123 (13.8)		744 (91.0)	74 (9.0)		250 (90.3)	27 (9.7)	
2	832 (86.2)	133 (13.8)		355 (82.0)	78 (18.0)		393 (87.9)	54 (12.1)		145 (91.2)	14 (8.8)	
≥3	1,270 (81.6)	286 (18.4)		669 (78.7)	181 (21.3)		528 (87.3)	77 (12.7)		15 (83.1)	31 (16.9)	
HIV test			15.29^§^			0.17			26.06^§^			2.39
Yes	1991 (87.47)	285 (12.52)		819 (82.81)	170 (17.18)		970 (92.29)	81 (7.7)		362 (89.82)	41 (10.17)	
No	1763 (83.31)	353 (16.68)		975 (82.13)	212 (17.86)		695 (84.85)	124 (15.14)		185 (85.64)	31 (14.35)	
Income			21.7^§^			5.1*			29.01^§^			4.39
<3,000	375 (85.4)	64 (14.6)		132 (83.0)	27 (17.0)		194 (84.3)	36 (15.7)		62 (93.9)	4 (6.1)	
3,000–5,000	965 (81.5)	219 (18.5)		508 (79.6)	130 (20.4)		439 (84.3)	82 (15.7)		56 (82.4)	12 (17.6)	
>5,000	2,412 (87.2)	354 (12.8)		1,152 (83.7)	224 (16.3)		1,032 (92.2)	87 (7.8)		429 (88.5)	56 (11.5)	
Length of residence			1.5			0.07			8.32^§^			0.33
0–6 months	579 (84.4)	107 (15.6)		228 (82.3)	49 (17.7)		306 (85.0)	54 (15.0)		75 (89.3)	9 (10.7)	
6–24 months	576 (86.7)	88 (13.3)		225 (83.0)	46 (17.0)		295 (88.6)	38 (11.4)		79 (89.8)	9 (10.2)	
>24 months	2,594 (85.4)	442 (14.6)		1,336 (82.4)	286 (17.6)		1,064 (90.4)	113 (9.6)		393 (87.9)	54 (12.1)	
Sexual orientation			5.485			4.9			3.18			3.05
Homosexual	2,632 (85.0)	463 (15.0)		1,194 (82.0)	262 (18.0)		1,211 (88.3)	161 (11.7)		402 (87.6)	57 (12.4)	
Heterosexual	67 (90.5)	7 (9.5)		47 (90.4)	5 (9.6)		18 (90)	2 (10)		3 (100)	0 (0.0)	
Bisexual	780 (85.2)	135 (14.8)		418 (81.5)	95 (18.5)		315 (91.3)	30 (8.7)		98 (88.3)	13 (11.71)	
Unsure	273 (89.2)	33 (10.8)		133 (86.9)	20 (13.1)		121 (91.0)	12 (9.0)		44 (95.7)	2 (4.3)	
Sexual roles			39.92^§^			11.48^§^			30.28^§^			1.77
Inserters	1,207 (90.5)	127 (9.5)		526 (86.5)	82 (13.5)		558 (94.9)	30 (5.1)		202 (90.6)	21 (9.4)	
Inserted	681 (82.4)	145 (17.6)		291 (78.9)	78 (21.1)		336 (85.9)	55 (14.1)		101 (86.3)	16 (13.7)	
Both	1819 (83.5)	360 (16.5)		928 (81.1)	216 (18.9)		771 (86.5)	120 (13.5)		244 (87.5)	35 (12.5)	
Preferred platform to find male sex partners			7.3^§^			6.64^§^			1.56			0.87
Venues	721 (83.0)	148 (17.0)		275 (78.1)	77 (21.9)		229 (87.4)	33 (12.6)		88 (90.7)	9 (9.3)	
Internet	2,277 (85.6)	382 (14.4)		1,108 (82.7)	232 (17.3)		941 (89.8)	107 (10.2)		383 (88.2)	51 (11.6)	
Others	756 (87.5)	108 (12.5)		411 (84.9)	73 (15.1)		495 (88.4)	65 (11.6)		76 (86.4)	12 (13.6)	
STIs			15.53^§^			2.4			45.17^§^			0.14
Yes	2,824 (86.9)	427 (13.1)		190 (78.8)	51 (21.2)		289 (79.2)	76 (20.8)		32 (86.5)	5 (13.5)	
No	930 (81.5)	211 (18.5)		1,604 (82.9)	331 (17.1)		1,376 (91.4)	129 (8.6)		515 (88.5)	67 (11.5)	
MB			4.28^§^			0.24			0.528			2.65
Yes	180 (80.7)	43 (19.3)		146 (81.1)	34 (18.9)		22 (84.6)	4 (15.4)		8 (72.7)	3 (27.3)	
No	3,574 (85.7)	595 (14.3)		1,648 (82.6)	348 (17.4)		1,643 (89.1)	201 (10.9)		539 (88.7)	69 (11.3)	
MSMW			0.45			0.92			0.43			0.001^§^
No	3,344 (85.3)	574 (14.7)		1,515 (82.1)	330 (17.9)		1,547 (88.9)	193 (11.1)		516 (88.4)	68 (11.7)	
Yes	410 (86.5)	64 (13.5)		279 (84.3)	52 (15.7)		118 (90.8)	12 (9.2)		31 (88.6)	4 (11.4)	
UAI			27.91^§^			1.67			20.67^§^			16.19^§^
Yes	1,326 (81.8)	295 (18.2)		697 (81.1)	162 (18.9)		553 (84.6)	101 (15.4)		160 (80.8)	38 (19.2)	
No	2,428 (87.6)	343 (12.4)		1,097 (83.3)	220 (16.7)		1,112 (91.4)	104 (8.6)		387 (91.9)	34 (8.1)	
HIV services			1.59			0.49			6.95			0.94
Yes	2,698 (85.9)	443 (14.1)		1,307 (82.1)	285 (17.9)		1,173 (90.3)	126 (9.7)		409 (89.1)	50 (10.9)	
No	1,056 (84.4)	195 (15.6)		487 (83.4)	97 (16.6)		492 (86.2)	79 (13.8)		138 (86.2)	22 (13.8)	
Core MSM			0.22			1.22			0.01			0.1
Yes	946 (85.8)	156 (14.2)		440 (80.9)	104 (19.1)		419 (89.1)	51 (10.9)		135 (87.7)	19 (12.3)	
No	2,819 (85.3)	487 (14.7)		1,354 (83.0)	278 (17.0)		1,246 (89.0)	154 (11.0)		412 (88.6)	53 (11.4)	
Seek sexual partners in core venues			5.19^§^			4.75			1.16			0.16
No	2,521 (86.3)	401 (13.7)		1,158 (83.8)	224 (16.2)		1,259 (88.6)	162 (11.4)		341 (88.8)	43 (11.2)	
Yes	1,244 (83.7)	242 (16.3)		636 (80.1)	158 (19.9)		406 (90.4)	43 (9.6)		206 (87.7)	29 (12.3)	
HIV nearby			5.5^§^			1.97			0.37			0.785
No	2,834 (86.2)	454 (13.8)		1,284 (83.2)	260 (16.8)		1,369 (89.2)	165 (10.8)		449 (88.9)	56 (11.1)	
Yes	919 (83.3)	184 (16.7)		508 (80.6)	122 (19.4)		296 (88.1)	40 (11.9)		98 (86.0)	16 (14.0)	
Non-HIV test nearby			2.2			0.062			0.005			0.024
No	1,475 (86.5)	231 (13.5)		584 (82.1)	127 (17.9)		743 (89.0)	92 (11.0)		294 (88.5)	38 (11.4)	
Yes	2,278 (84.8)	407 (15.2)		1,208 (82.6)	255 (17.4)		922 (89.1)	113 (10.9)		253 (88.2)	34 (11.8)	
MB nearby			3.59*			0.081			2.87*			0.098
No	3,465 (85.8)	575 (14.2)		1,567 (82.5)	332 (17.5)		1,631 (89.2)	197 (10.8)		535 (88.4)	70 (11.6)	
Yes	288 (82.1)	63 (17.9)		225 (81.8)	50 (18.2)		34 (81.0)	8 (19.0)		12 (85.7)	2 (14.3)	
MSMW nearby			0.008			2.03			0.37			7.24^§^
No	3,077 (85.4)	524 (14.6)		1,316 (81.7)	294 (18.3)		1,479 (88.9)	185 (11.1)		502 (89.5)	59 (10.5)	
Yes	676 (85.6)	114 (14.4)		476 (84.4)	88 (15.6)		186 (90.3)	20 (9.7)		45 (77.6)	13 (22.4)	
UAI nearby			1.49			2.43			4.1^§^			0.61
No	1,727 (86.2)	277 (13.8)		487 (84.5)	89 (15.5)		556 (91.1)	54 (8.9)		285 (89.3)	34 (10.7)	
Yes	2,026 (84.9)	361 (15.1)		1,305 (81.7)	293 (18.3)		1,109 (88.0)	151 (12.0)		262 (87.3)	38 (12.7)	
Drug abuse nearby			3.46^§^			0.03			3.119*			0.61
No	2,831 (84.9)	503 (15.1)		1,600 (82.5)	340 (17.5)		1,074 (88.1)	145 (12.0)		285 (89.3)	34 (10.7)	
Yes	922 (87.2)	135 (12.8)		192 (82.1)	42 (17.9)		591 (90.8)	60 (9.2)		262 (87.3)	38 (12.7)	
Age^#^	30.1 (7.8)	30.5 (8.7)	0.951	29.0 (7.2)	29.18 (7.57)	0.24	30.82 (7.9)	31.51 (9.2)	1.45	32.4 (8.4)	33.5 (9.7)	1.06

^#^Analysis of variance (ANOVA). ^§^*p* < 0.05; *p < 0.1.

**Table 2:** A comparative analysis of the demographic and behavioral characteristics of MSM who had sought sexual partners in core venues and who had not.

	Overall (2010–2022)			2010–2014			2015–2019			2020–2022		
	No (*n* = 2,922)	Yes (*n* = 1,486)	*χ*^2^/*F*	No (*n* = 1,382)	Yes (*n* = 794)	*χ*^2^/*F*	No (*n* = 1,421)	Yes (*n* = 449)	*χ*^2^/*F*	No (*n* = 384)	Yes (*n* = 235)	*χ*^2^/*F*
PLWHA												
No	2,521 (86.3)	1,244 (83.7)	5.19*	1,158 (83.8)	636 (80.1)	4.75*	1,259 (88.6)	406 (90.4)	1.16	341 (88.8)	206 (87.7)	0.19
Yes	401 (13.7)	242 (16.3)		224 (16.2)	158 (19.9)		162 (11.4)	43 (9.6)		43 (11.2)	29 (12.3)	
Educational levels			9.05*			1.34			10.81*			1.82
Junior high school and below	364 (12.5)	196 (13.2)		206 (14.9)	120 (15.1)		158 (11.1)	56 (12.5)		28 (7.3)	20 (8.5)	
High middle school	774 (26.5)	451 (30.3)		484 (35.0)	295 (37.1)		288 (20.3)	121 (26.9)		69 (18.0)	51 (21.7)	
College and above	1,771 (60.6)	834 (56.1)		692 (50.1)	377 (47.5)		975 (68.6)	272 (60.6)		287 (74.7)	164 (69.8)	
UAI			1.32			7.69*			0.32			0.25
Yes	1,091 (37.337)	530 (35.666)		576 (41.678)	283 (35.642)		492 (34.623)	162 (36.080)		120 (31.25)	78 (33.191)	
No	1818 (62.217)	953 (64.131)		806 (58.321)	511 (64.357)		929 (65.376)	287 (63.919)		264 (68.75)	157 (66.808)	
Drug abuse			0.97			4.44*			0.12			0.21
Yes	377 (12.9)	208 (14.0)		66 (4.8)	55 (6.9)		318 (22.4)	97 (21.6)		81 (21.1)	46 (19.6)	
No	2,532 (86.7)	1,275 (85.8)		1,316 (95.2)	739 (93.1)		1,103 (77.6)	352 (78.4)		303 (78.9)	189 (80.4)	
HIV test			105.03**			40.46**			56.11**			34.91**
Yes	1,347 (46.1)	929 (62.5)		557 (40.3)	432 (54.4)		730 (51.4)	321 (71.5)		216 (56.3)	187 (79.6)	
No	1,562 (53.5)	554 (37.3)		825 (59.7)	362 (45.6)		691 (48.6)	128 (28.5)		168 (43.8)	48 (20.4)	
HIV service			168.13**			78.38**			67.9**			15.4**
Yes	1,897 (64.9)	1,244 (83.7)		923 (66.8)	669 (84.3)		917 (64.5)	382 (85.1)		264 (68.8)	195 (83.0)	
No	1,012 (34.6)	239 (16.1)		459 (33.2)	125 (15.7)		504 (35.5)	67 (14.9)		120 (31.3)	40 (17.0)	
Income			27.54**			23.91**			2.85			3.07
<3,000	333 (11.4)	106 (7.1)		119 (8.6107)	40 (5.0377)		185 (13.019)	45 (10.022)		47 (12.239)	19 (8.0851)	
3,000–5,000	809 (27.7)	375 (25.2)		438 (31.7)	200 (25.2)		392 (27.6)	129 (28.7)		39 (10.2)	29 (12.3)	
>5,000	1,766 (60.4)	1,000 (67.3)		824 (59.6)	552 (69.5)		844 (59.4)	275 (61.2)		298 (77.6)	187 (79.6)	
Length of residence			23.11**			1.03			12.49*			9.45*
0–6 months	500 (17.111)	186 (12.516)		174 (12.590)	103 (12.972)		296 (20.830)	64 (14.253)		62 (16.145)	22 (9.3617)	
6–24 months	462 (15.811)	202 (13.593)		180 (13.024)	91 (11.460)		260 (18.296)	73 (16.258)		61 (15.885)	27 (11.489)	
>24 months	1,946 (66.598)	1,090 (73.351)		1,028 (74.384)	594 (74.811)		865 (60.872)	312 (69.487)		261 (67.968)	186 (79.148)	
Sexual orientation			24.93**			31.55**			1.6			3.43
Homosexual	2,057 (70.4)	1,038 (69.9)		938 (67.9)	518 (65.2)		1,042 (73.3)	330 (73.5)		280 (72.9)	179 (76.2)	
Heterosexual	30 (1.0)	44 (3.0)		14 (1.0)	38 (4.8)		16 (1.1)	4 (0.9)		1 (0.3)	2 (0.9)	
Bisexual	605 (20.7)	310 (20.9)		327 (23.7)	186 (23.4)		257 (18.1)	88 (19.6)		76 (19.8)	35 (14.9)	
Unsure	217 (7.4)	89 (6.0)		103 (7.5)	50 (6.3)		106 (7.5)	27 (6.0)		27 (7.0)	19 (8.1)	
Sexual roles			9.28*			0.78			14.36**			0.72
Inserters	839 (28.7)	495 (33.3)		374 (27.1)	234 (29.5)		416 (29.3)	172 (38.3)		142 (37.0)	81 (34.5)	
Inserted	568 (19.4)	258 (17.4)		233 (16.9)	136 (17.1)		315 (22.167)	76 (16.926)		74 (19.3)	43 (18.3)	
Both	1,457 (49.9)	722 (48.6)		728 (52.7)	416 (52.4)		690 (48.6)	201 (44.8)		168 (43.8)	111 (47.2)	
Preferred platform to find male sex partners			207.96**			59.1**			386.89**			26.31**
Venues	396 (13.6)	473 (31.8)		160 (11.8)	192 (24.2)		73 (5.1)	189 (42.1)		38 (9.9)	59 (25.1)	
Internet	1,884 (64.5)	775 (52.2)		895 (64.8)	445 (56.0)		876 (61.6)	172 (38.3)		284 (74.0)	150 (63.9)	
Others	629 (21.5)	235 (15.8)		327 (23.7)	157 (19.8)		472 (33.2)	88 (19.6)		62 (16.1)	26 (11.1)	
MB			52.18**			38.38**			7.09*			0.27
Yes	98 (3.4)	125 (8.4)		76 (5.5)	104 (13.1)		14 (1.0)	12 (2.7)		6 (1.6)	5 (2.1)	
No	2,811 (96.2)	1,358 (91.4)		1,306 (94.5)	690 (86.9)		1,407 (99.0)	437 (97.3)		378 (98.4)	230 (97.9)	
STIs			6.65*			3.44			2.41			0.51
Yes	271 (9.3)	175 (11.8)		140 (10.1)	101 (12.7)		266 (18.7)	99 (22.0)		25 (6.5)	12 (5.1)	
No	2,638 (90.3)	1,308 (88.0)		1,242 (89.9)	693 (87.3)		1,155 (81.3)	350 (78.0)		359 (93.5)	223 (94.9)	
Number of sexual partners			12.56*			1.64			16.55**			0.31
1	1,268 (43.4)	603 (40.6)		580 (42.0)	313 (39.4)		632 (44.5)	186 (41.4)		175 (45.6)	102 (43.4)	
2	663 (22.7)	302 (20.3)		275 (19.9)	158 (19.9)		362 (25.5)	85 (18.9)		98 (25.5)	61 (26.0)	
≥3	978 (33.5)	578 (38.9)		527 (38.1)	323 (40.7)		427 (30.0)	178 (39.6)		111 (28.9)	72 (30.6)	
MSMW			0.91			0.08			4.34*			0.07
Yes	2,606 (89.2)	1,312 (88.3)		1,174 (84.9)	671 (84.5)		1,332 (93.7)	408 (90.9)		363 (94.5)	221 (94.0)	
No	316 (10.8)	175 (11.8)		208 (15.1)	123 (15.5)		89 (6.3)	41 (9.1)		21 (5.5)	14 (6.0)	
Core MSM			1.68			0.6			2.86			0.76
No	2,174 (74.4)	1,133 (76.2)		1,029 (74.5)	603 (76.0)		343 (24.1)	127 (28.1)		91 (23.7)	63 (26.8)	
Yes	748 (25.6)	354 (23.8)		353 (25.5)	191 (24.1)		1,078 (75.9)	325 (71.9)		293 (76.3)	172 (73.2)	
Age^#^	29.6 (7.7)	31.4 (8.4)	53.23**	28.5 (6.9)	29.9 (7.9)	17.88**	30.1 (7.9)	32.7 (8.7)	32.56**	31.7 (8.3)	33.8 (8.8)	8.46*

^#^Variance analysis. *p < 0.05; **p < 0.001.

As listed in Table 3, the PLWHA in the SCN were more likely to have ≥3 sexual partners (aOR = 1.47, 95% CI: 1.11–1.79), a sexual role was receptive (aOR = 2.04, 95% CI: 1.57–2.64) or both (aOR = 1.81, 95% CI: 1.45–2.25), had sought sexual partners in core venues (aOR = 1.30, 95% CI: 1.08–1.56), and drug abuse (aOR = 1.41, 95% CI: 1.11–1.79) and were less likely to have a high middle school education level (aOR = 0.71, 95% CI: 0.55–0.91) or college or above educational level (aOR = 0.48, 95% CI: 0.38–0.61), had been testing HIV (aOR = 0.68, 95% CI: 0.57–0.81) and non-UAI (aOR = 0.70, 95% CI: 0.59–0.84).

**Table 3:** Logistic regression analyses of characteristics of PLWHA and non-PLWHA.

	Overall (2010–2022)		2010–2014		2015–2019		2020–2022	
	aOR	95%CI	aOR	95%CI	aOR	95%CI	aOR	95%CI
Educational levels								
Junior high school and below	ref.		ref.		ref.			
High middle school	0.71	0.55–0.91	0.69	0.50–0.94	0.75	0.48–1.18		
College and above	0.48	0.38–0.61	0.54	0.39–0.73	0.40	0.26–0.61		
Number of sexual partners								
1	ref.							
2	1.17	0.92–1.48	1.38	1.00–1.89				
≥3	1.47	1.11–1.79	1.59	1.23–2.06				
Sexual roles								
Inserters	ref.				ref.			
Inserted	2.04	1.57–2.65	1.74	1.23–2.45	3.03	1.87–4.91		
Both	1.81	1.46–2.25	1.43	1.09–1.89	2.75	1.79–4.21		
Seek sexual partners in core venues								
Yes	1.30	1.08–1.56	1.28	1.02–1.61				
No	ref.		ref.					
Drug abuse								
Yes	1.41	1.11–1.79			1.43	1.01–2.02	2.09	1.21–3.61
No	ref.				ref.		ref.	
HIV test								
Yes	0.68	0.57–0.81			0.48	0.35–0.66		
No					ref.			
STIs								
Yes					2.42	1.74–3.36		
No					ref.			
UAI								
No	0.70	0.59–0.84			0.58	0.42–0.78	0.4	0.24–0.66
Yes	ref.				ref.		ref.	
MSMW nearby								
No							ref.	
Yes							2.51	1.25–5.02

As listed in Table 4, MSM who had sought sexual partners in core venues were more likely to be PLWHA (aOR = 1.30, 95% CI: 1.08–1.57), older (aOR = 1.02, 95% CI: 1.01–1.03), sexual orientation was heterosexual (aOR = 2.62, 95% CI: 1.54–4.44), average monthly income >5,000 (aOR = 1.61, 95% CI: 1.25–2.08), were MB (aOR = 2.44, 95% CI: 1.78–3.34), had been testing HIV (aOR = 1.77, 95% CI: 1.53–2.03), and had been receiving HIV intervention services (aOR = 2.14, 95% CI: 1.80–2.53) and were less likely were floating residents (length of residence in Shenzhen is 0–6 months) (aOR = 0.72, 95% CI: 0.58–0.88), seeking sexual partners on the Internet (aOR = 0.34, 95% CI: 0.29–0.41), or others (aOR = 0.33, 95% CI: 0.27–0.41).

**Table 4:** Logistic regression analyses of characteristics of MSM who had sought sexual partners in core venues and who had not been.

	Overall (2010–2022)		2010–2014		2015–2019		2020–2022	
	aOR	95% CI	aOR	95% CI	aOR	95% CI	aOR	95% CI
PLWHA								
No	Ref.		Ref.					
Yes	1.30	1.08–1.57	1.31	1.03–1.67				
HIV test								
No	Ref.		Ref.		Ref.		Ref.	
Yes	1.77	1.53–2.03	1.568	1.29–1.90	2.44	1.85–3.22	3.08	2.05–4.63
HIV service								
No	Ref.		Ref.		Ref.		Ref.	
Yes	2.14	1.80–2.53	2.17	1.71–2.75	2.17	1.57–3.06	1.69	1.09–2.64
Income								
<3,000	Ref.		Ref.					
3,000–5,000	1.16	0.89–1.53	1.01	0.66–1.54				
>5,000	1.61	1.25–2.08	1.70	1.14–2.54				
Length of residence								
0–6 months	Ref.				Ref.			
6–24 months	0.72	0.58–0.88			0.61	0.42–0.87		
>24 months	0.83	0.68–1.01			0.91	0.65–1.28		
Sexual orientation								
Homosexual	Ref.		Ref.					
Heterosexual	2.62	1.54–4.44	3.81	1.89–7.69				
Bisexual	0.99	0.83–1.17	0.98	0.79–1.23				
Unsure	0.89	0.67–1.17	0.94	0.65–1.37				
Preferred platform to find male sex partners								
Venues	Ref.		Ref.		Ref.		Ref.	
Internet	0.34	0.29–0.41	0.42	0.33–0.55	0.068	0.049–0.096	0.30	0.19–0.49
Others	0.33	0.27–0.41	0.43	0.32–0.58	0.070	0.048–0.10	0.23	0.12–0.44
MB								
No	Ref.		Ref.					
Yes	2.44	1.78–3.34	2.13	1.47–3.08				
Age	1.02	1.01–1.03	1.03	1.01–1.04	1.02	1.01–1.04	1.03	1.01–1.05
Drug abuse								
No			Ref.					
Yes			1.57	1.06–2.33				
UAI								
No			Ref.					
Yes			0.79	0.65–0.96				

### Subgroup analysis

Among 2,176, 1,889, and 619 MSM in the periods 2010–2014, 2015–2019, and 2020–2022, 17.6% (382/2176), 12.3% (230/1889), and 11.6% (72/619) of whom were PLWHA, respectively (Appendix Figures 2–4); and 60, 29, and 21 MSM venues for the three time periods were enrolled in the MSM venues network; among which 15, 8, and 6 MSM venues were defined as core venues, and 794 (36.5%, 794/2176), 449 (23.8%, 449/1889), and 235 (38.0%, 235/619) MSM who had sought sexual partners in the core venues were reported in the periods 2010–2014, 2015–2019, and 2020–2022, respectively (Appendix Table 1). The demographic and behavioral characteristics comparative analysis among PLWHA and non-PLWHA are listed in Table 1, and among MSM who had sought sexual partners in core venues and MSM who had not sought sexual partners in core venues are listed in Table 2.

As shown in Table 4, during the period 2010–2014, MSM who had sought sexual partners in core venues were associated with a higher risk of HIV than those who did not (aOR = 1.31, 95% CI: 1.03–1.67). However, from the periods 2015–2019 and 2020–2022, no statistically significant variations in HIV prevalence were observed between MSM who had sought sexual partners in core venues and those who did not. Notably, the PLWHA were more likely to have multiple sexual partners, a lower educational level, a UAI, a sexual role was receptive or both, drug abuse, and infected with STIs (Table 3). Across all yearly subgroups, a consistent observation is that MSM who had sought sexual partners in core venues were significantly more likely to be older, have undergone HIV testing and accessed HIV intervention services within the past 12 months, and less likely to seek sexual partners through the Internet or other mediums (Table 4).

### Assortative

As listed in Table 5, the sexual contact network exhibited minimal assortative among MSM who had sought sexual partners in core venues, PLWHA, UAI, HIV test, HIV service, length of residence, MSMW, sexual orientation, sexual roles, preferred platform to find male sex partners, STIs. However, assortative was only observed among the core MSM and MB.

**Table 5:** Assortativity of nodes in sexual contact networks.

	Overall (2010–2022)	2010–2014	2015–2019	2020–2022
PLWHA	0.03	0.03	0.06	0.03
UAI	0.01	−0.01	0.01	0.05
HIV test	0.04	0.01	0.05	−0.04
HIV service	0.02	0.02	0.02	0.03
Length of residence	0.03	0.02	0.02	0.04
MSMW	0.07	0.04	0.08	0.05
Core MSM	0.85	0.46	0.53	0.38
MSM who seek sexual partners in core venues	0.05	0.04	0.04	−0.01
Drug abuse	0.02	0.02	0.002	−0.02
Sexual orientation	0.02	0.03	0.001	−0.03
Sexual roles	−0.01	−0.001	−0.01	−0.04
MB	0.13	0.1	0.27	−0.01
Preferred platform to find male sex partners	0.03	0.04	0.02	−0.02
STIs	0.02	0.003	0.003	0.04

## Discussion

By leveraging the SCN to delve into the factors driving HIV transmission among MSM, we can gain profound insights that inform the development of tailored and innovative HIV intervention strategies. The findings of this study revealed that the HIV prevalence among MSM in the SCN (11.6%–17.6%) was higher than the local MSM during the same period (5%–10% in sentinel surveillance data). This suggested that HIV was predominantly transmitted within the larger SCN, and MSM who are part of this extensive network may face a significantly elevated risk of contracting the virus compared to those outside it. Therefore, SCN-based expanded HIV testing strategies could be highly effective in increasing the diagnostic efficiency of identifying PLWHA among MSM (15). Such targeted approaches have the potential to significantly reduce HIV transmission within this key population and contribute to the overall effort to contain the epidemic (16).

Previous studies have suggested that the HIV-related risk behaviors of other members of the SCN were important influencing factors of the risk of HIV among MSM (9, 17). However, that was not found to be the result in this study, and MSM whose neighboring nodes were MSMW were found to have a higher risk of HIV only in 2020–2022. It may be due to the absence of clear predispositions for MSM sexual contact. We calculated assortative coefficients for HIV risks among MSM in SCN, which indicated a tendency for sexually active MSM to associate with like-minded individuals, yet other risks exhibited minimal assortative. Especially considering the influential factor of sexual role in the risk of HIV (18), the analyses indicate that while MSM disclosed their sexual role preferences in the survey, these may not have translated into distinct categorizations during actual sexual encounters, suggesting potential ambiguity in practice. This neutral network poses challenges for precision interventions, as MSM SCN lack clear categorical markers, thereby hindering the development of tailored strategies that could effectively address individual risk profiles.

In this study, the MSM venue network was created based on MSM venues population movement data. Therefore, the core venues in the venue network should be the most prominent MSM seeking sexual partners. Prior research indicated that MSM venues frequented by MSM could facilitate unprotected sexual activity, thereby significantly contributing to the elevated risk of HIV transmission within this community (19). MSM who engage in venue-based activities have been identified as a subgroup within the MSM that faces a particularly high risk of contracting HIV (6, 7). The results of this study revealed that the MSM who had sought sexual partners in core venues had a higher risk of HIV than those who had not in 2010–2014. After implementation of the venue-based differentiated behavioral interventions, which promote joint marketing between the CDC and MSM venues across all venues within Shenzhen, commencing in 2015 (20, 21). There were no statistically significant variations in HIV prevalence between the MSM who had sought sexual partners in the core venues and those who had not, in 2015–2019 and 2020–2022. This absence of a significant disparity may be largely attributable to the efficacy of the venue-based differentiated behavioral interventions.

Before introducing the venue-based differentiated behavioral interventions, the CDC had been carrying out routine interventions on the premises for an extended period. Although the MSM who had sought sexual partners in core venues were more likely to have non-UAI, HIV tests and access to HIV services, they nonetheless continued to have higher risk of HIV compared to the MSM who had not, in 2010–2014. This could be due to the MSM who had sought sexual partners in core venues were more likely were drug abuse which could increase the risk of HIV (22). It suggested that curbing the transmission and dissemination of HIV among MSM remains challenging without a holistic approach to mitigating their HIV-related risk behaviors. Moreover, the high HIV prevalence in core venues, coupled with the fact that a lot of PLWHA did not receive ART (Immediate ART implemented in 2016) from 2010 to 2014 (23), likely resulted in numerous potential sources of HIV in core venues, which may contribute to an increased risk of HIV. Therefore, the ART strategy also helps to explain the declined prevalence in 2015–2019 and 2020–2022.

Our study revealed that the Internet was the primary medium for MSM to seek sexual partners, with even over 50% of those who had sought sexual partners in core venues reporting they preferred to seek sexual partners via the Internet. However, the proportion of those who had received HIV service among MSM who had sought sexual partners in the core venues was significantly higher than the MSM who had not been. The disparity in accessing HIV services could be attributed to the uneven allocation of public health resources currently. Prior research has indicated that MSM are increasingly relying on MSM-specific APPs to find sexual partners (24). Furthermore, a study conducted in Shenzhen from 2015 to 2017 revealed that MSM who used these APPs to seek sexual partners tended to exhibit multiple sexual partners and UAI (25). While substantial investment in public health resources directed toward MSM venues could contribute to mitigating the risk of HIV among MSM who had sought sexual partners in MSM venues, it is imperative that we meticulously consider adjustments to the allocation of these resources. We need to acknowledge the evolving needs and dynamics within MSM communities. Augmenting public health resources dedicated to HIV services for MSM who seek sexual partners on the Internet to enhance their accessibility is crucial to mitigate HIV-related risk behaviors and ultimately decreasing incidence rates.

Shenzhen is the city with the most significant number of floating residents in China. Previous investigations have suggested that in Shenzhen, MSM are more likely to be floating residents (length of residence in Shenzhen is 0–6 months) (1), and the HIV epidemiology has been predominantly driven by floating residents (26).The result of this study suggested that MSM who had sought sexual partners in core venues were less likely to be floating residents, which implies that the MSM who were floating residents could be inclined to utilize the Internet or alternative channels to seek sexual partners in Shenzhen. Prior research has indicated that MSM were increasingly relying on MSM-specific APPs to seek sexual partners, thus reducing their dependence on MSM venues for this purpose (24). Therefore, in Shenzhen, while the implementation of the venue-based differentiated behavioral interventions could effectively mitigate HIV among MSM who had sought sexual partners in core venues, their influence on the floating residents could be constrained.

### Limitations

Our study has several limitations. First, the limited number of referrals per respondent in the RDS survey may have led to underestimating the respondent’s influence on the SCN. However, we tried to reduce the impact of respondents in the SCN of MSM as much as possible by adding virtual nodes. Second, inadequate sampling of the floating residents in RDS surveys may have introduced bias, necessitating the development of advanced methodologies to capture a comprehensive MSM sexual contact network in the future research studies. Third, given the extended duration of the entire study, the influence of some individuals within the network might have been distorted due to their replication over time. Nevertheless, we addressed this concern by conducting subgroup analyses, specifically tailored to the unique context of Shenzhen and the year of the survey, thereby minimizing the potential bias introduced by repeated individuals and enhancing the robustness of our results.

## Conclusion

In Shenzhen, MSM who had sought sexual partners in core venues had a higher risk of HIV compared to those not. After 2015, venue-based differentiated behavioral interventions mitigated this disparity, eliminating significant differences in the risk of HIV. Given substantial initial HIV intervention investments in MSM venues, leading to inequitable access for MSM who had sought sexual partners in venues and who had not, the resource allocation strategy for HIV intervention services necessitates prompt adjustment to curb HIV transmission among MSM.

## Data Availability

The datasets used and/or analyzed during the current study are available from the corresponding author (Jin Zhao) upon reasonable request.
