# Supplementary material for: HIV risk and influence factors among MSM who had sought sexual partners in core venues: a continuous sentinel surveillance in 2010–2022
Source: Front Public Health. 2024 Dec 16;12:1476642. doi: 10.3389/fpubh.2024.1476642 (PMC11683098; doi:10.3389/fpubh.2024.1476642)
Supplement: Supplementary file 1 [file Table_1.doc]

| **Appendix Table 1. network degree and HIV Prevalence in MSM venues in the sexual contact network of MSM.** | | | | | | | | | | | | |
| --- | --- | --- | --- | --- | --- | --- | --- | --- | --- | --- | --- | --- |
| **Name** | **Overall (2010-2022)** | | | **2010-2014** | | | **2015-2019** | | | **2020-2022** | | |
|  | **Degree** | **HIV-prevalence** | **Core venues** | **Degree** | **HIV-prevalence** | **Core venues** | **Degree** | **HIV-prevalence** | **Core venues** | **Degree** | **HIV-prevalence** | **Core venues** |
| Bar 1 | 296 | 14.1 | Yes | 154 | 20.1 | Yes | 101 | 5.9 | Yes | 53 | 13.2 | Yes |
| Bar 2 | 140 | 16.4 | Yes | 135 | 15.6 | Yes | 5 | 40.0 | No | - | - | - |
| Bar 3 | 166 | 10.8 | Yes | 134 | 11.2 | Yes | 29 | 10.3 | No | 7 | 0.0 | No |
| Bar 4 | 297 | 13.8 | Yes | 132 | 18.9 | Yes | 87 | 9.2 | Yes | 86 | 11.6 | Yes |
| Bar 5 | 185 | 19.4 | Yes | 127 | 26.0 | Yes | 45 | 11.1 | Yes | 18 | 5.6 | No |
| Bar 6 | 191 | 16.7 | Yes | 120 | 20.8 | Yes | 54 | 13.0 | Yes | 24 | 12.5 | Yes |
| Bar 7 | 125 | 22.4 | Yes | 91 | 23.1 | Yes | 31 | 16.1 | Yes | 5 | 40.0 | No |
| Bar 8 | 99 | 14.1 | Yes | 34 | 17.6 | Yes | 48 | 12.5 | Yes | 20 | 10.0 | Yes |
| Bar 9 | 21 | 19 | No | 21 | 19.0 | No | - | - | - | - | - | - |
| Bar 10 | 18 | 11.1 | No | 16 | 12.5 | No | 2 | 0.0 | No | - | - | - |
| Bar 11 | 11 | 18.1 | No | 9 | 11.1 | No | 2 | 50.0 | No | - | - | - |
| Bar 12 | 6 | 0 | No | 6 | 0.0 | No | - | - | - | - | - | - |
| Bar 13 | 7 | 28.5 | No | 4 | 25.0 | No | 3 | 33.3 | No | - | - | - |
| Bar 14 | 3 | 0 | No | 3 | 0.0 | No | - | - | - | - | - | - |
| Bar 15 | 15 | 6.66 | No | 15 | 6.7 | Yes | - | - | - | - | - | - |
| Bar 16 | 4 | 50 | No | 4 | 50.0 | No | - | - | - | - | - | - |
| Bar 17 | 3 | 0 | No | 3 | 0.0 | No | - | - | - | - | - | - |
| Bar 18 | 1 | 0 | No | 1 | 0.0 | No | - | - | - | - | - | - |
| Bar 19 | 178 | 11.2 | Yes | - | - | - | 32 | 9.4 | No | 146 | 11.6 | Yes |
| Bar 20 | 100 | 10 | Yes | - | - | - | 30 | 10.0 | No | 73 | 9.6 | Yes |
| Bar 21 | 7 | 0 | No | - | - | - | 6 | 0.0 | No | 1 | 0.0 | No |
| Bar 22 | 10 | 10 | No | - | - | - | 6 | 0.0 | No | 4 | 25.0 | No |
| Club 1 | 53 | 18.8 | Yes | 52 | 19.2 | Yes | - | - | - | 1 | 0.0 | No |
| Club 2 | 30 | 10 | No | 24 | 12.5 | Yes | 4 | 0.0 | No | 2 | 0.0 | No |
| Club 3 | 19 | 10.5 | No | 13 | 0.0 | No | 5 | 40.0 | No | 1 | 0.0 | No |
| Club 4 | 10 | 40 | No | 10 | 40.0 | No | - | - | - | - | - | - |
| Club 5 | 10 | 20 | No | 10 | 20.0 | No | - | - | - | - | - | - |
| Club 6 | 8 | 25 | No | 8 | 25.0 | No | - | - | - | - | - | - |
| Club 7 | 6 | 0 | No | 6 | 0.0 | No | - | - | - | - | - | - |
| Club 8 | 4 | 50 | No | 4 | 50.0 | No | - | - | - | - | - | - |
| Club 9 | 3 | 33.3 | No | 3 | 33.3 | No | - | - | - | - | - | - |
| Club 10 | 3 | 33.3 | No | 3 | 33.3 | No | - | - | - | - | - | - |
| Club 11 | 1 | 0 | No | 1 | 0.0 | No | - | - | - | - | - | - |
| Club 12 | 1 | 0 | No | 1 | 0.0 | No | - | - | - | - | - | - |
| Club 13 | 1 | 0 | No | 1 | 0.0 | No | - | - | - | - | - | - |
| Club 14 | 11 | 18.1 | No | 11 | 18.2 | No | - | - | - | - | - | - |
| Club 15 | 7 | 14.2 | No | 7 | 14.3 | No | - | - | - | - | - | - |
| Club 16 | 6 | 33.3 | No | 6 | 33.3 | No | - | - | - | - | - | - |
| Club 17 | 4 | 25 | No | 4 | 25.0 | No | - | - | - | - | - | - |
| Club 18 | 3 | 33.3 | No | 3 | 33.3 | No | - | - | - | - | - | - |
| Club 19 | 3 | 0 | No | 3 | 0.0 | No | - | - | - | - | - | - |
| Club 20 | 2 | 50 | No | 2 | 50.0 | No | - | - | - | - | - | - |
| Club 21 | 2 | 50 | No | 2 | 50.0 | No | - | - | - | - | - | - |
| Club 22 | 2 | 0 | No | 2 | 0.0 | No | - | - | - | - | - | - |
| Club 23 | 2 | 0 | No | 2 | 0.0 | No | - | - | - | - | - | - |
| Club 24 | 2 | 50 | No | - | - | - | 2 | 50.0 | No | - | - | - |
| Saunas 1 | 511 | 19.1 | Yes | 289 | 24.2 | Yes | 226 | 13.7 | No | 16 | 12.5 | No |
| Saunas 2 | 139 | 24.4 | No | 130 | 24.6 | No | 9 | 44.4 | No | 3 | 33.3 | No |
| Saunas 3 | 43 | 23.2 | No | 42 | 23.8 | No | 1 | 100.0 | No | - | - | - |
| Saunas 4 | 23 | 34.7 | Yes | 21 | 33.3 | Yes | 2 | 50.0 | Yes | - | - | - |
| Saunas 5 | 17 | 29.4 | Yes | 17 | 29.4 | Yes | - | - | - | - | - | - |
| Saunas 6 | 14 | 14.2 | Yes | 13 | 15.4 | Yes | - | - | - | 1 | 0.0 | No |
| Saunas 7 | 12 | 8.33 | Yes | 12 | 8.3 | Yes | - | - | - | 1 | 0.0 | No |
| Saunas 8 | 12 | 25 | No | 9 | 22.2 | No | 3 | 33.3 | No | - | - | - |
| Saunas 9 | 38 | 21 | No | 8 | 37.5 | No | 29 | 20.7 | No | 2 | 0.0 | No |
| Saunas 10 | 5 | 20 | No | 5 | 20.0 | No | - | - | - | - | - | - |
| Saunas 11 | 4 | 25 | No | 4 | 25.0 | No | - | - | - | - | - | - |
| Saunas 12 | 2 | 50 | No | 2 | 50.0 | No | - | - | - | - | - | - |
| Saunas 13 | 4 | 25 | No | 4 | 25.0 | No | - | - | - | - | - | - |
| Park 1 | 28 | 32.1 | No | 28 | 35.7 | No | - | - | - | - | - | - |
| Park 2 | 23 | 34.7 | No | 23 | 34.8 | No | - | - | - | - | - | - |
| Park 3 | 6 | 16.6 | No | 6 | 16.7 | No | - | - | - | - | - | - |
| Other 1 | 12 | 25 | No | 12 | 25.0 | No | - | - | - | - | - | - |
| Other 2 | 7 | 14.2 | No | 5 | 0.0 | No | 2 | 50.0 | No | - | - | - |
| Other 3 | 16 | 25 | No | 3 | 33.3 | No | 8 | 37.5 | No | 5 | 0.0 | No |
| Other 4 | 15 | 13.3 | No | - | - | - | 15 | 13.3 | No | - | - | - |
| Other 5 | 2 | 100 | No | - | - | - | 2 | 100.0 | No | - | - | - |
| Other 6 | 1 | 0 | No | - | - | - | - | - | - | 1 | 0.0 | No |
