# Supplementary figures and images for: HIV risk and influence factors among MSM who had sought sexual partners in core venues: a continuous sentinel surveillance in 2010–2022
Source: Front Public Health. 2024 Dec 16;12:1476642. doi: 10.3389/fpubh.2024.1476642 (PMC11683098; doi:10.3389/fpubh.2024.1476642)

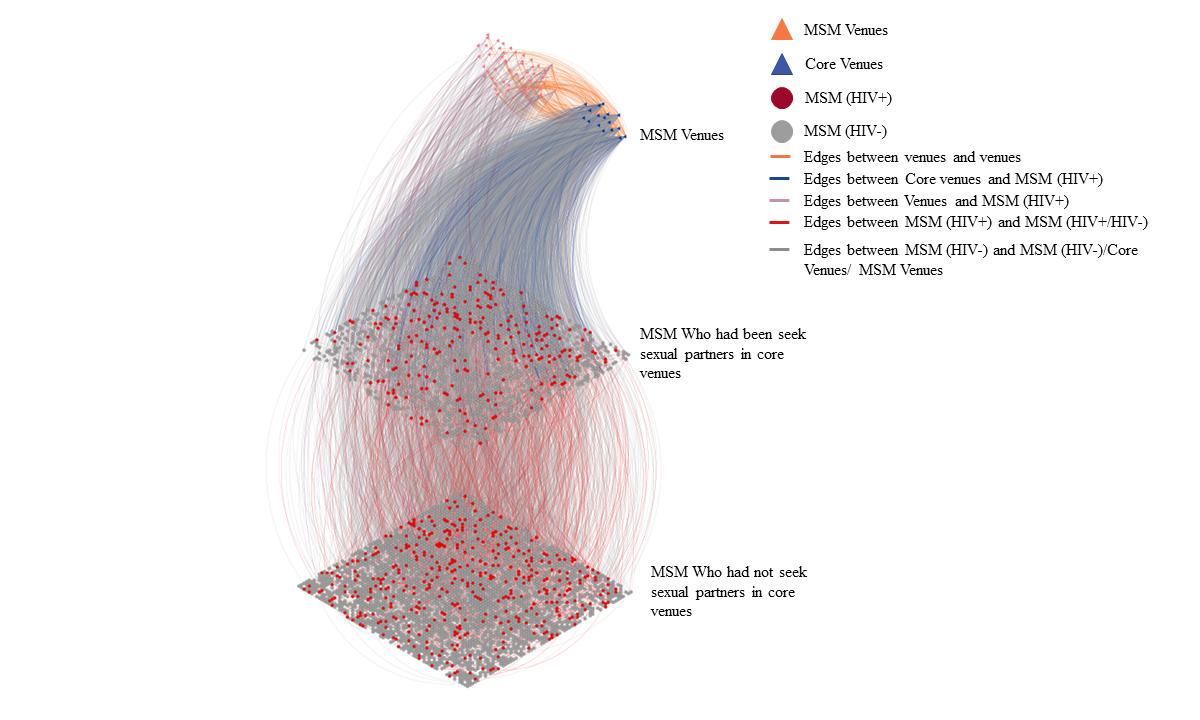

Supplement: Supplementary file 2 [file Image_1.png]

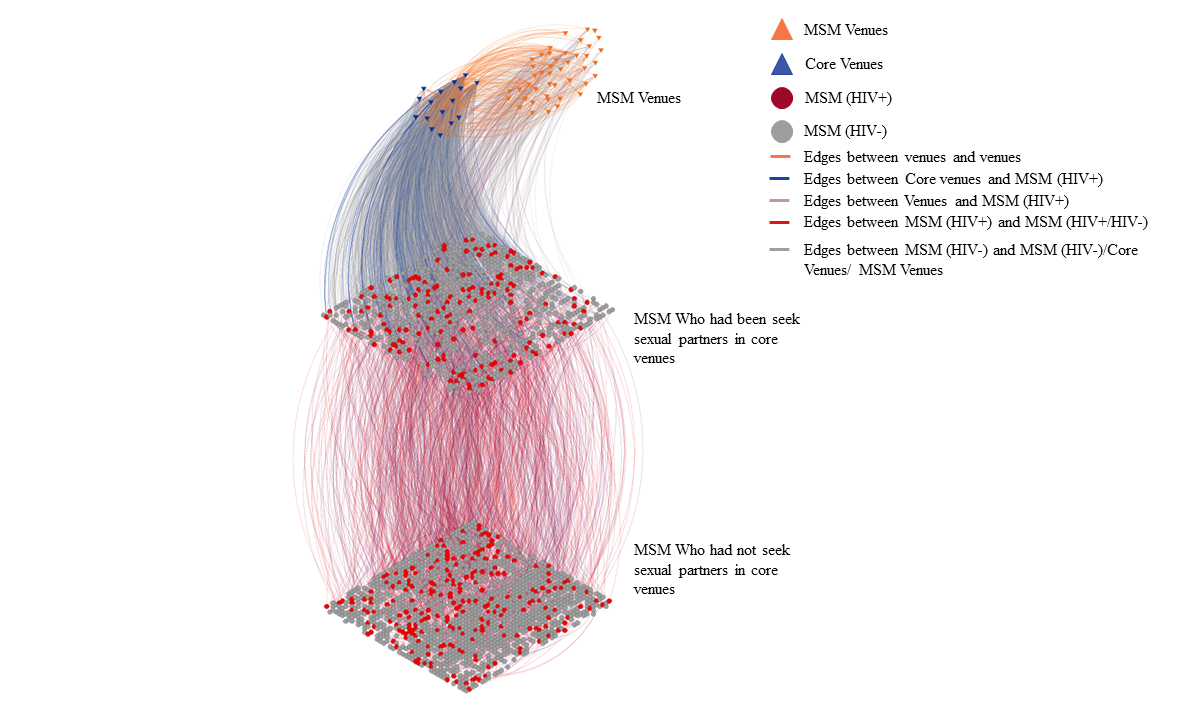

Supplement: Supplementary file 3 [file Image_2.png]

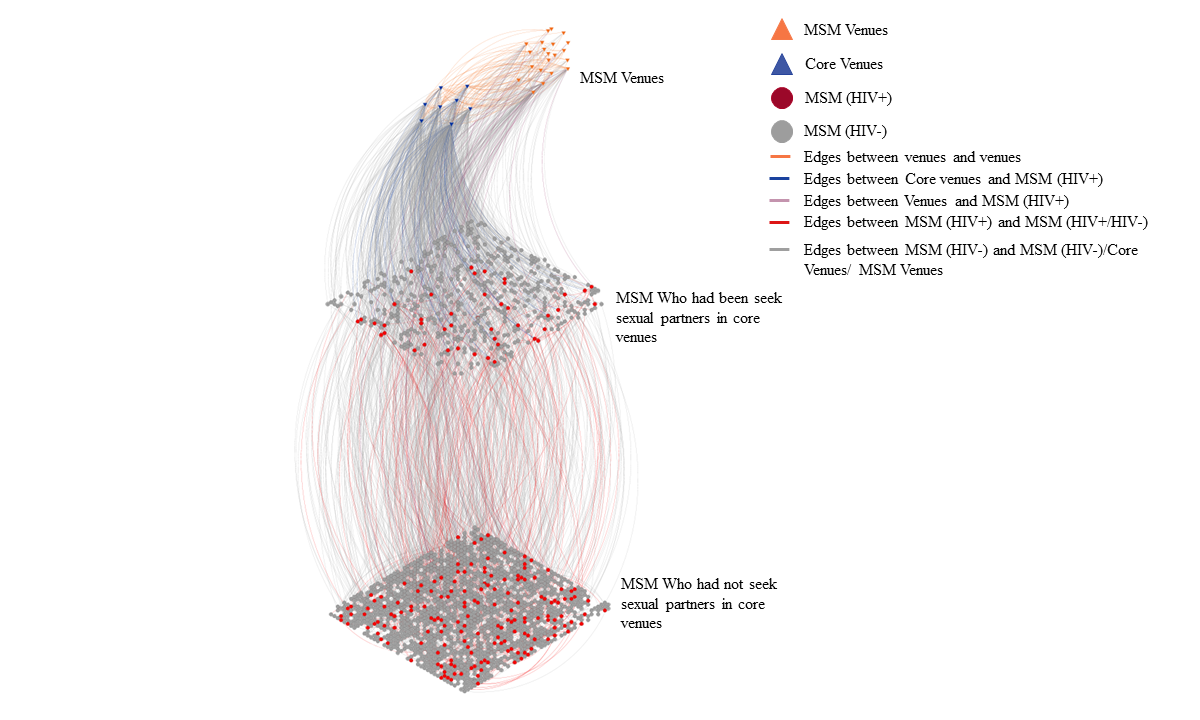

Supplement: Supplementary file 4 [file Image_3.png]

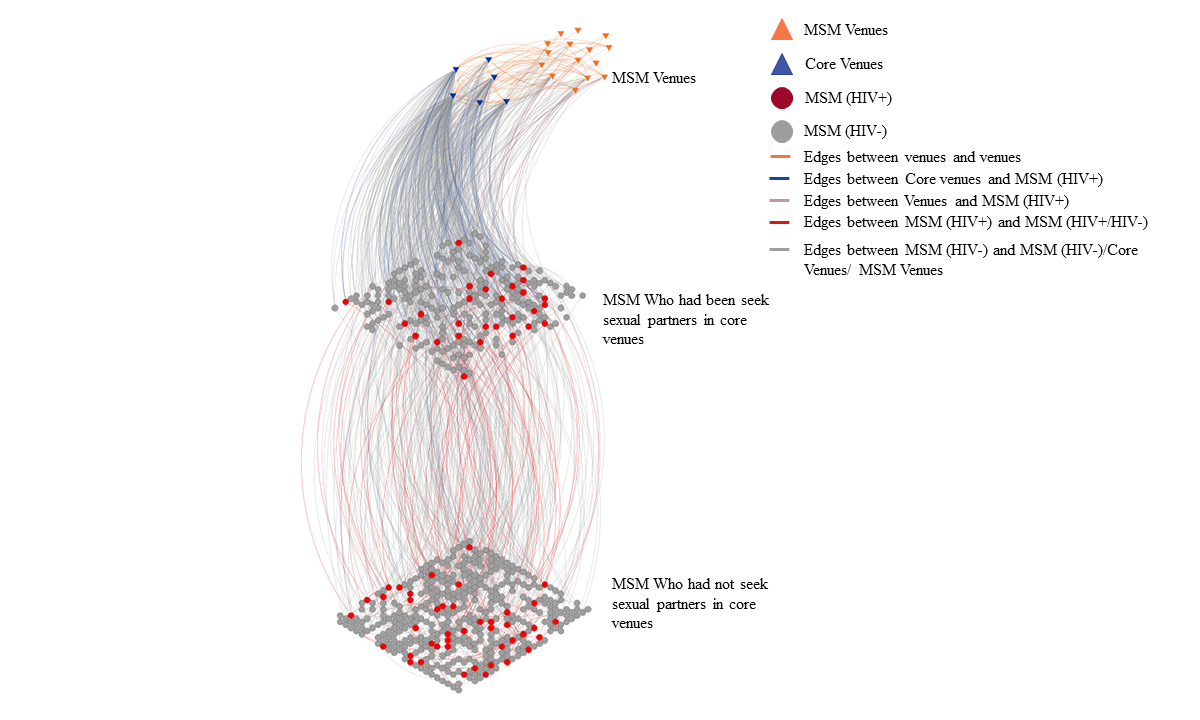

Supplement: Supplementary file 5 [file Image_4.png]
